# Effects of long COVID on healthcare utilization

**DOI:** 10.1371/journal.pone.0327218

**Published:** 2025-07-23

**Authors:** Michael Gottlieb, Erica S. Spatz, Huihui Yu, Imtiaz Ebna Mannan, Michelle Santangelo, Caitlin Malicki, Nicole L. Gentile, Rachel E. Geyer, Alex Charlton, Jonathan W. Dyal, Joann G. Elmore, Kristyn Gatling, Mandy J. Hill, Juan Carlos C. Montoy, Kelli N. O’Laughlin, Kristin L. Rising, Sharon Saydah, Kari A. Stephens, Ralph C. Wang, Lauren E. Wisk, Arjun K. Venkatesh, Robert A. Weinstein, for the Innovative Support for Patients with SARS-CoV-2 Infections Registry (INSPIRE) Group

**Affiliations:** 1 Department of Emergency Medicine, Rush University Medical Center, Chicago, Illinois, United States of America; 2 Section of Cardiovascular Medicine, Yale School of Medicine, New Haven, Connecticut, United States of America; 3 Department of Epidemiology, Yale School of Medicine, New Haven, Connecticut, United States of America; 4 Yale Center for Outcomes Research and Evaluation, Yale School of Medicine, New Haven, Connecticut, United States of America; 5 Department of Emergency Medicine, Yale School of Medicine, New Haven, Connecticut, United States of America; 6 Department of Family Medicine, University of Washington, Seattle, Washington, United States of America; 7 Department of Laboratory Medicine and Pathology, University of Washington, Seattle, Washington, United States of America; 8 Center for Connected Care, Thomas Jefferson University, Philadelphia, Pennsylvania, United States of America; 9 Department of Emergency Medicine, Johns Hopkins University, Baltimore, Maryland, United States of America; 10 Division of General Internal Medicine & Health Services Research, David Geffen School of Medicine at UCLA, Los Angeles, California, United States of America; 11 Division of Infectious Diseases, Department of Medicine, Rush University Medical Center, Chicago, Illinois, United States of America; 12 Department of Population Health and Health Disparities, University of Texas Medical Branch at Galveston, Galveston, Texas, United States of America; 13 Department of Emergency Medicine, University of California - San Francisco, San Francisco, California, United States of America; 14 Departments of Emergency Medicine and Global Health, University of Washington, Seattle, Washington, United States of America; 15 Department of Emergency Medicine, Sidney Kimmel Medical College, Thomas Jefferson University, Philadelphia, Pennsylvania, United States of America; 16 National Center for Immunizations and Respiratory Diseases, Centers for Disease Control & Prevention, Atlanta, Georgia, United States of America; 17 Department of Biomedical Informatics & Medical Education, University of Washington, Seattle, Washington, United States of America; 18 Division of Infectious Diseases, Department of Medicine, Cook County Hospital, Chicago, Illinois, United States of America; University of Oxford, UNITED KINGDOM OF GREAT BRITAIN AND NORTHERN IRELAND

## Abstract

**Background:**

While most research on Long COVID (LC) has focused on symptoms and quality of life, there remains a critical need to better understand the effect of LC on resource utilization. This study sought to determine the type and amount of healthcare utilization among participants with versus without LC.

**Methods:**

This was a secondary analysis of a prospective, longitudinal, multicenter U.S. study of adult participants with symptomatic COVID-19, confirmed with testing, who completed 3-month post-infection surveys and had electronic health record data for at least 180 days pre- and post-index testing. We excluded participants with any COVID-19 infections within the 6 months following enrollment. Consistent with prior work, LC was defined as ≥3 post-infectious symptoms at 3 months, while those with <3 symptoms were categorized as not having LC. Our primary outcome was to compare the change in visit types between pre- and post-index testing (hospitalization, emergency department visit, office visit, procedure, telehealth, and other). As secondary outcomes, we assessed differences in visit complexity using the summative length of each encounter for each category as a measure of total healthcare usage.

**Results:**

A total of 847 participants met inclusion criteria (179 LC, 668 non-LC). When compared with the pre-index period, there was an overall increase in visit numbers of all six visit categories during the post-index period for all groups, most pronounced in office and telehealth visits. When compared with the non-LC group, the LC group was less likely to have ED visits (OR: 0.1; 95% CI 0.0–0.5). However, among those with LC who had at least one hospitalization, they were more likely to have additional hospitalizations (OR: 2.6; 95% CI 1.5–4.6). Visit length for office visits and hospitalization in the LC group was increased when compared with the non-LC group, though this diminished after adjustment for patient baseline characteristics.

**Conclusions:**

All participants who were infected with SARS-CoV-2 had a marked increase in healthcare utilization during the subsequent 180 days. The LC group had significantly higher rates of additional hospitalization compared with those without LC, which may help to inform healthcare resource planning.

## Introduction

Since late 2019, severe acute respiratory syndrome coronavirus 2 (SARS-CoV-2) has spread globally with over 776 million reported cases [1]. An important subset of these patients develop persistent symptoms lasting at least 4–12 weeks, commonly referred to as Long COVID (LC) [2–4]. LC can have a profound impact on health status, quality of life, and return to work or other activities [5–9]. Though exact prevalence estimates vary, LC poses a significant public health burden.

While many studies of the costs and resource utilization associated with SARS-CoV-2 infection have focused on the acute period, data on longer-term resource utilization associated with persistent symptoms are limited. An early estimate from 2020 projected that the societal impact of LC may approximate $2.6 trillion [10]. Subsequent studies seeking to validate projections have relied primarily on insurance claims data. One study using the IQVIA PharMetrics Plus database found that patients with COVID-19 had a $892 increase in total cost of care at 6 months post-infection compared with people without COVID-19 [11]. A cohort study in Israel using the Maccabi Healthcare Services database found that patients with LC had a 1.76 to 2.47 adjusted odds ratio for subsequent hospitalization and increases in healthcare expenditures compared with those without LC [12]. A separate study reported an estimated 415.8 excess total visits per 1,000 patients for select post-COVID conditions [13]. While these studies underscore the financial implications of LC, they often focus on cost or simply report a total number of care encounters without delving into the specific types of healthcare encounters or the broader aspects of resource utilization. Our understanding of how persistent post-infectious symptoms influence healthcare usage remains incomplete, particularly regarding the frequency and duration of different healthcare services.

This study aims to examine healthcare utilization patterns among adults tested for SARS-CoV-2 who exhibited acute symptoms at the time of testing. By leveraging data from a prospective, multicenter study that combined self-reported outcomes and electronic health record (EHR) linkage, we can compare healthcare utilization between SARS-CoV-2 positive participants with and without LC. This approach allows us to provide a more nuanced understanding of the long-term healthcare needs of individuals affected by LC.

## Methods

### Study design and population

This was a secondary analysis of a multicenter U.S. study of adult participants tested for SARS-CoV-2 to assess healthcare utilization (Clinicaltrials.gov NCT04610515). The Innovative Support for Patients with SARS-CoV-2 Infections Registry (INSPIRE) study is a prospective, longitudinal study conducted across eight major healthcare systems selected for diversity of geography and patient population. Sites recruited participants broadly, irrespective of primary state of residence, to maximize geographic dispersion of the participants. Participants were prospectively enrolled if they had symptoms consistent with acute COVID-19 infection and tested either positive or negative for SARS-CoV-2. Participants were enrolled either virtually or in-person across eight study sites, including Rush University (Chicago, Illinois; enrolled 11/17/2020–8/22/2022), Yale University (New Haven, Connecticut; enrolled 12/26/2020–8/22/2022), the University of Washington (Seattle, Washington; enrolled 12/11/2020–8/22/2022), Thomas Jefferson University (Philadelphia, Pennsylvania; enrolled 2/8/2021–8/22/2022), the University of Texas Southwestern (Dallas, Texas; enrolled 4/21/2021–8/22/2022), the University of Texas, Houston (Houston, Texas; enrolled 5/5/2021–8/22/2022), the University of California, San Francisco (San Francisco, California; enrolled 2/24/2021–8/22/2022) and the University of California, Los Angeles (Los Angeles, California; enrolled 2/1/2021–8/22/2022). All participants electronically signed a written informed consent. They were followed longitudinally, completing surveys every three months for a maximum of 18 months. Participants were also asked to share access to their EHR via a secure digital platform (Hugo Health, LLC; Guilford, CT). For this analysis, the index test date range was November 16, 2020 – July 29, 2022; the three-month surveys were completed between March 16, 2021 – November 3, 2022. The study was approved by the institutional review boards at all eight institutions (Rush University Institutional Review Board #20030902-IRB01; Thomas Jefferson University Institutional Review Board #20P.1150; University of California Los Angeles Institutional Review Board #20−001683; University of California San Francisco Institutional Review Board #20−32222; UTHealth Committee for the Protection of Human Subjects #HSC-MS-20–0981; University of Texas Southwestern Medical Center Institutional Review Board #STU-2020–1352; University of Washington Institutional Review Board #STUDY00009920; Yale University Institutional Review Board #2000027976). This study follows the Strengthening the Reporting of Observational Studies in Epidemiology (STROBE) reporting guidelines for cohort studies [14].

The full study details have been published elsewhere [15]. Briefly, inclusion criteria included age ≥ 18 years, fluency in English or Spanish, reported symptoms of SARS-CoV-2 infection (e.g., fever, shortness of breath, cough), and completion of an antigen or polymerase chain reaction test for SARS-CoV-2 within the preceding 42 days. Exclusion criteria included being lawfully imprisoned, inability of the study team to confirm the result of the index diagnostic test for SARS-CoV-2, having a previous SARS-CoV-2 infection >42 days before enrollment, and lacking access to an internet-connected device (e.g., smartphone, tablet, computer) for electronic survey completion. Additionally, participants were required to complete their baseline survey within 42 days of their index SARS-CoV-2 test date and connect at least one EHR system to Hugo Health (required through March 21, 2022). For this analysis, we excluded participants without SARS-CoV-2 infection on initial testing, with a second SARS-CoV-2 infection within six months following the index SARS-CoV-2 test, and those without access to EHR data for at least 180 days before and after the index SARS-CoV-2 test. We collected symptom data informed by the Centers for Disease Control and Prevention Person Under Investigation symptom list, which included symptoms commonly reported at the time of the survey (e.g., fever, fatigue, headache, loss of smell).

Consistent with prior work, we defined LC as a participant who was COVID-positive with ≥3 post-infectious symptoms at 3 months (e.g., fatigue, dyspnea, anosmia) [6,8,16,17]. While a wide range of definitions exist, this definition was selected *a priori* and has been consistently used across all INSPIRE studies performed at that time. A participant was categorized as not having LC (non-LC) if they were COVID-positive but did not have at least three post-infectious symptoms at 3 months.

### Health care utilization outcomes

We extracted the information (e.g., type, date, and time of admission and discharge) in the “encounter” component from the EHR data extracted in the Consolidated Clinical Document Architecture (C-CDA) format. We categorized the available healthcare encounters into six categories: inpatient hospitalization, emergency department visit, office visit (regardless of specialty), procedure, telehealth visit (regardless of specialty), and other. Categorization was performed based on careful consideration of available information related to the encounters (e.g., encounter codes, encounter name) by independent review from five investigators (MG, ESS, IEM, AKV, RAW) with any discrepancies resolved via discussion and consensus.

Our primary outcome was the number of encounters across each category at the pre- and post-index periods. We also assessed visit complexity using total hours of healthcare service utilization stratified by each encounter type at the pre- and post-index periods. We defined the ‘pre-index’ period as the 180 days prior to the index SARS-CoV-2 test. We defined the ‘post-index’ period as between 30–180 days after the index SARS-CoV-2 test. We excluded the first 0–29 days from analysis as we deemed this more likely to be related to the acute illness as opposed to long-term effects of the infection. The upper limit of 180 days post-index was selected to maximize the capture of the number and duration of encounters after the infection.

For each participant, we counted the number of unique visits (based on admission and discharge time, location, and services received) and summed the length of encounter as the visit complexity of each encounter type at pre- and post-index periods. For calculating the length of each encounter, we applied the Winsorization approach to handle encounters with an atypical length [18,19]. Specifically, the length of office and telehealth visits were Winsorized at the 85^th^ percentile (1.0 and 1.5 hours, respectively); the length of the other four types of encounters was Winsorized at the 95^th^ percentile. For analyzing the difference in length between cohorts, we excluded visits categorized within the procedure and ‘other’ groups as there were multiple confounding differences in these categories (e.g., difference in time required for procedures, heterogeneity in visits included within the ‘other’ group).

### Statistical analysis

We compared key socio-demographics and baseline health status between LC and non-LC. We conducted chi-square tests for categorical variables and the Kruskal-Wallis test for age, which was a continuous variable with a non-normal distribution. To assess the change of healthcare usage within each cohort, we compared the observed pre- and post-index prevalence of participants with each type of encounter via testing the equality of proportions. We also presented the distribution of the frequency and length of encounter among participants with each type of encounter at pre- and post-index test periods. Further, to assess the difference in the changes of healthcare utilization among the two cohorts, we conducted zero-inflated Poisson (ZIP) regression models to estimate the difference in the probability of having a given type of encounter and the probability of having one more encounter among participants with that type of encounter [20,21]. We also employed generalized linear regression models (GLMs) to assess the difference in the change of total hours of healthcare usage for each type of encounter among the two cohorts. Further, for both ZIP models and GLMs, we added participant demographics (age and gender), viral variant at the index COVID test, baseline symptom severity scores, and eight baseline medical conditions (i.e., asthma, hypertension, diabetes, obesity, COPD, heart conditions, kidney disease, and liver disease) for adjustment. We presented unadjusted and adjusted odds ratios of having any encounters and incidence-rate ratio of having an additional encounter between the two cohorts estimated from the ZIP models. We also presented unadjusted and adjusted differences in hours of healthcare usage among the two cohorts that were estimated from the GLM models. The statistical analyses were conducted using SAS v9.4 (SAS Institute Inc) and R version 4.2.2 (R Foundation for Statistical Computing). All tests were 2-sided with a significance threshold of p-value = 0.05.

## Results

**Fig 1:**
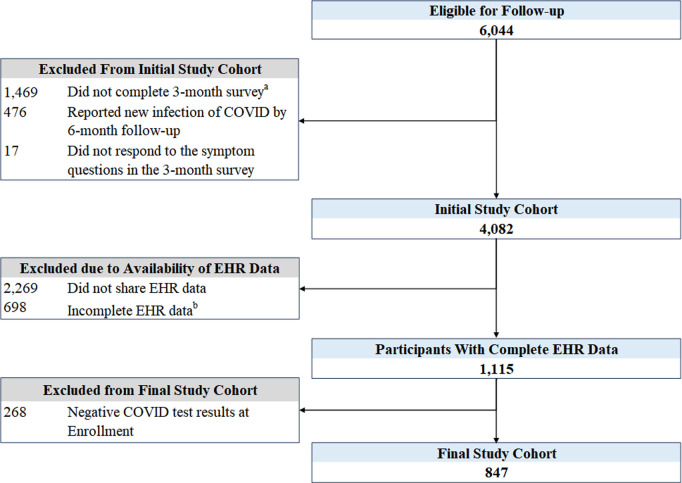
Flow diagram of study cohort. ^a^ Incomplete surveys due to withdrawal, deceased, and loss to follow-up. ^b^ Incomplete EHR data includes participants who shared EHR data but did not have EHR data + /-180 days from index test.

Of the 6,044 enrolled participants, 4,082 met initial eligibility criteria. Among those, 2,967 were excluded due to lack of access to EHR data and 268 due to a negative COVID test, with 847 meeting the final inclusion criteria (Fig 1). This included 179 in the LC group and 668 in the non-LC group. The participants had a median age of 40 years (Q1-Q3: 31–54). The majority were female (64.0%). Participants were more commonly non-Hispanic (86.7%), white (72.7%), had at least a 4-year college degree (70.9%), and were married/had a partner (59.1%). Obesity (27.7%), hypertension (16.3%), and asthma (12.5%) were the most frequently reported pre-existing conditions (Table 1).

**Table 1:** Participant characteristics by cohort.

Characteristics	Long COVID (N = 179)	No Long COVID (N = 668)	Total (N = 847)	*p*-value
**Age** ^a^				
Median (Q1-Q3)	42 (34–53)	39 (31–54)	40 (31–54)	.0760
**Categorical Variables, n (column %)**				
**Gender**				
Female	137 (80.1)	405 (62.0)	542 (64.0)	<0.0001
Male	32 (18.7)	238 (36.4)	270 (31.9)	
Transgender/Non-binary	2 (1.2)	10 (1.5)	12 (1.4)	
Missing	8 (4.5)	15 (2.2)	23 (2.7)	
**Ethnicity**				
Non-Hispanic	150 (86.7)	584 (88.5)	734 (86.7)	.1086
Hispanic	23 (13.3)	76 (11.5)	99 (11.7)	
Missing	6 (3.4)	8 (1.2)	14 (1.7)	
**Race**				
White	121 (69.1)	495 (75.2)	616 (72.7)	.0848
Black	22 (12.6)	52 (7.9)	74 (8.7)	
Asian	12 (6.9)	62 (9.4)	74 (8.7)	
Other/Multiple	20 (11.4)	49 (7.4)	69 (8.1)	
Missing	4 (2.2)	10 (1.5)	14 (1.7)	
**Education**				
Less than High school	1 (0.6)	3 (0.5)	4 (0.5)	.0034
High school graduate	14 (7.9)	34 (5.2)	48 (5.7)	
Some College	36 (20.3)	91 (13.8)	127 (15.0)	
2-year degree	17 (9.6)	38 (5.8)	55 (6.5)	
4-year degree	65 (36.7)	220 (33.4)	285 (33.6)	
More than 4 years	44 (24.9)	272 (41.3)	316 (37.3)	
Missing	2 (1.1)	10 (1.5)	12 (1.4)	
**Marital Status**				
Married/partner	99 (55.6)	402 (60.3)	501 (59.1)	.0109
Divorced/Widowed/Separated	29 (16.3)	55 (8.2)	84 (9.9)	
Never married	50 (28.1)	210 (31.5)	260 (30.7)	
Missing	1 (0.6)	1 (0.1)	2 (0.2)	
**Family Income**				
<10,000	12 (6.7)	31 (4.6)	43 (5.1)	.0026
10,000-34,999	21 (11.7)	53 (7.9)	74 (8.7)	
35,000-49,999	25 (14.0)	46 (6.9)	71 (8.4)	
50,000-74,999	29 (16.2)	91 (13.6)	120 (14.2)	
≥75,000	84 (46.9)	416 (62.3)	500 (59.0)	
Prefer not to answer	8 (4.5)	31 (4.6)	39 (4.6)	
**Health Insurance**				
Private only	119 (66.5)	513 (76.8)	632 (74.6)	.0227
Public only	46 (25.7)	116 (17.4)	162 (19.1)	
Private and public	10 (5.6)	21 (3.1)	31 (3.7)	
None	4 (2.2)	18 (2.7)	22 (2.6)	
**Employment**				
Employed, essential	144 (80.4)	547 (82.0)	691 (81.6)	.9075
Not employed	35 (19.6)	120 (18.0)	155 (18.3)	
Missing	0 (0.0)	1 (0.1)	1 (0.1)	
**Tobacco Use**				
Any tobacco use	22 (12.3)	75 (11.2)	97 (11.5)	.0150
No tobacco use	157 (87.7)	593 (88.8)	750 (88.5)	
**Pre-existing Conditions**				
Asthma	39 (23.9)	67 (10.3)	106 (12.5)	<.0001
Hypertension	38 (23.3)	100 (15.4)	138 (16.3)	.0159
Diabetes	10 (6.1)	38 (5.8)	48 (5.7)	.8888
Obesity	63 (38.7)	172 (26.5)	235 (27.7)	.0021
Emphysema/COPD	6 (3.7)	4 (0.6)	10 (1.2)	.0015
Heart conditions	8 (4.9)	21 (3.2)	29 (3.4)	.3019
Kidney disease	4 (2.5)	10 (1.5)	14 (1.7)	.4217
Liver disease	3 (1.8)	6 (0.9)	9 (1.1)	.3169
**Testing Location**				
At home testing kit	9 (5.1)	91 (13.7)	100 (11.8)	<.001
Clinic including urgent care	37 (20.9)	77 (11.6)	114 (13.5)	
Emergency department	17 (9.6)	26 (3.9)	43 (5.1)	
Hospital	21 (11.9)	80 (12.0)	101 (11.9)	
Other	14 (7.9)	35 (5.3)	49 (5.8)	
Tent/drive up testing site	79 (44.6)	355 (53.5)	434 (51.2)	
Missing	2 (1.1)	4 (0.6)	6 (0.7)	
**Baseline COVID-19 Vaccination Status** ^c^				
Vaccinated	104 (58.1)	516 (77.2)	620 (73.2)	<.001
Not vaccinated	69 (38.6)	135 (20.2)	204 (24.1)	
Missing	6 (3.3)	17 (2.5)	23 (2.7)	

LC, Long COVID; PAIC, Post-acute Illness Complications.

^a^ Age was the only continuous variable in this table, which was reported with median and the interquartile range (Q1-Q3) and compared across cohorts based on Kruskal-Wallis test.

^b^ Hospitalization for index illness was a new question added to the 3-month Follow-up Survey beginning 4-14-2021.

^c^ SARS-CoV-2 vaccination status indicates participants with at least one dose prior to the index COVID-19 test; Vaccination initiation information was obtained from linked electronic health record data and patient survey responses.

Comorbidity questions were only asked on the 3-month follow-up survey beginning 04-14-2021. All p-values were calculated using chi-square tests except for age which was based on Kruskal-Wallis test. PAIC, Post-acute Illness Complications.

Compared with the non-LC group, those with LC were more likely to be female, have lower total household income, have asthma or chronic obstructive pulmonary disease, have obesity, and have not been vaccinated. A comparison of the participants included in study with all eligible participants is shown in S1 Table. Compared to included participants, those meeting eligibility criteria but excluded due to lack of access to EHR data were more likely to be younger, Asian, never married, use tobacco, and have fewer comorbidities.

**Fig 2:**
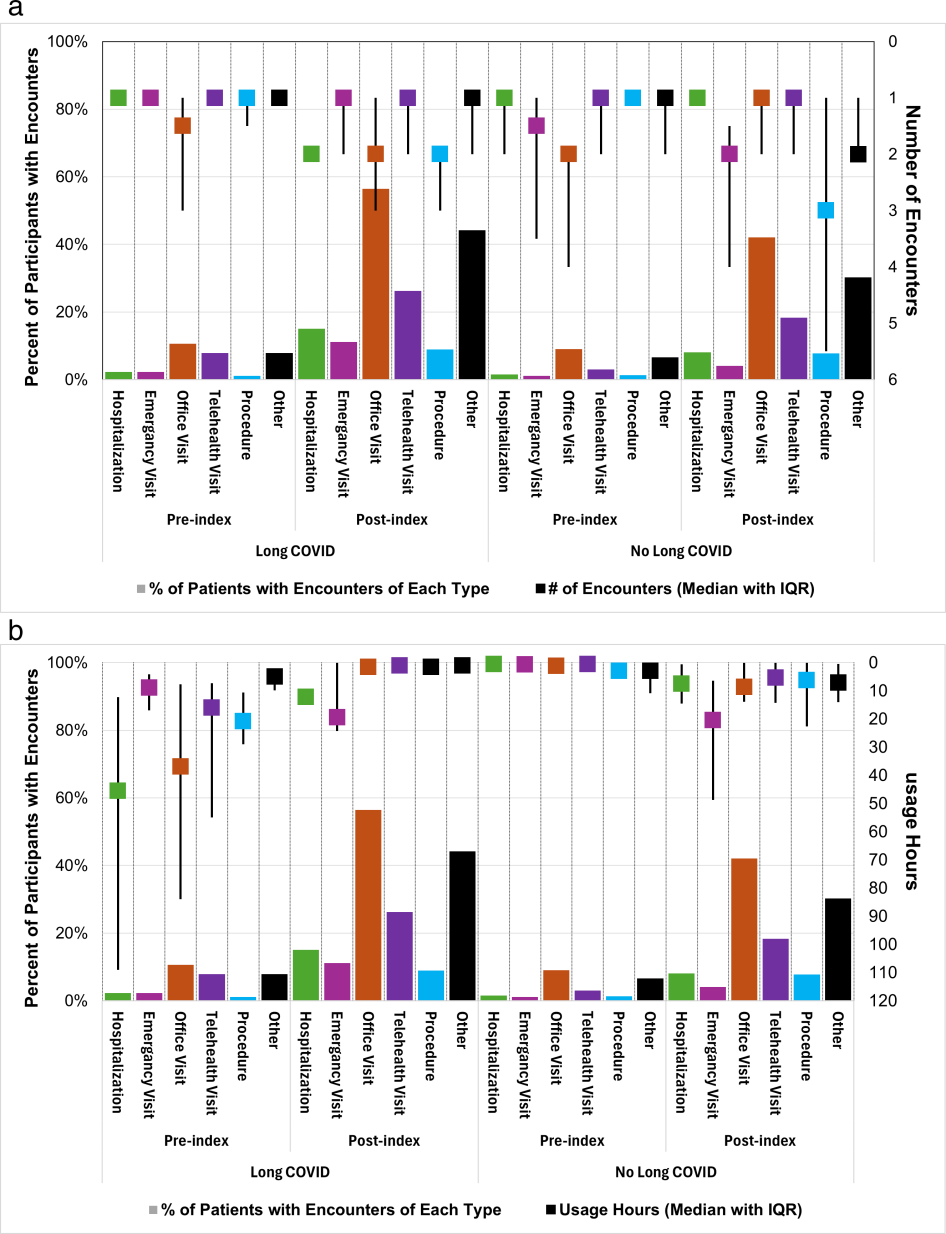
Observed healthcare utilization at pre- and post-index periods across the four cohorts. A, Percent of participants with each encounter type and the median number of encounters among those participants. B, Percent of participants with encounter type and the usage hours among those participants.

When assessing the proportion of participants with encounters during the pre-index versus post-index outcome windows, we saw a general increase in the number of all encounters across both the LC and non-LC cohorts (Table 2, Fig 2). Among those with LC, hospitalizations increased from 2.2% to 15.1% (p < 0.0001), emergency visits increased from 1.5% to 8.1% (p = 0.0007), office visits increase from 10.6% to 56.4% (p < 0.0001), telehealth increased from 7.8% to 26.3% (p < 0.0001), and procedures increased from 1.1% to 8.9% (p = 0.0007).

**Table 2:** Observed Healthcare Utilization at Pre- and Post-index Period.

Encounter Type	Cohort	N	Participants with the Type of Encounter						*p*-value ($\frac{n_{1}}{N}$ vs $\frac{n_{2}}{N}$)
			Pre-Index Period			Post-index Period			
			n_1_ (%)	# of Encounters^a^	Total Service Hours^b^	n_2_ (%)	# of Encounters^c^	Total Service Hours^d^	
				Median (Q1-Q3)^e^	Median (Q1-Q3)^e^		Median (Q1-Q3)^e^	Median (Q1-Q3)^e^	
Hospitalization	Long COVID	179	4 (2.2%)	1 (1 - 1)	45.5 (12.3 - 109)	27 (15.1%)	1 (1 - 2)	15 (10 - 28.4)	<.0001
	No Long COVID	668	10 (1.5%)	1 (1 - 1)	8.95 (4.2 - 16.9)	54 (8.1%)	1 (1 - 1)	13.65 (5.5 - 26.9)	<.0001
Emergency Visit	Long COVID	179	4 (2.2%)	1 (1 - 1.5)	20.8 (10.65 - 28.95)	20 (11.2%)	1 (1 - 2)	2.75 (1.3 - 10.1)	.0007
	No Long COVID	668	7 (1.0%)	1 (1 - 1)	4.9 (2.3 - 9.9)	27 (4.0%)	1 (1 - 1)	4.4 (2.5 - 11.6)	.0005
Office Visit^f^	Long COVID	179	19 (10.6%)	2 (1 - 3)	1.5 (0.7 - 2.2)	101 (56.4%)	2 (1 - 4)	1.5 (0.8 - 2.9)	<.0001
	No Long COVID	668	60 (9.0%)	1 (1 - 2)	1.1 (0.5 - 1.6)	281 (42.1%)	2 (1 - 3)	1.2 (0.7 - 2.1)	<.0001
Telehealth Visit^f^	Long COVID	179	14 (7.8%)	1 (1 - 2)	0.55 (0.3 - 1)	47 (26.3%)	2 (1 - 3)	0.5 (0.3 - 1.2)	<.0001
	No Long COVID	668	20 (3.0%)	1.5 (1 - 3.5)	0.7 (0.3 - 2.55)	122 (18.3%)	1 (1 - 3)	0.5 (0.2 - 1.3)	<.0001
Procedure	Long COVID	179	2 (1.1%)	1 (1 - 1)	3 (2.5 - 3.5)	16 (8.9%)	1 (1 - 1)	0.8 (0.4 - 8.2)	.0007
	No Long COVID	668	9 (1.3%)	1 (1 - 2)	2.9 (1 - 10.9)	52 (7.8%)	1 (1 - 2)	2.9 (0.95 - 10.5)	<.0001
Other	Long COVID	179	14 (7.8%)	1 (1 - 2)	8.6 (0.1 - 13.9)	79 (44.1%)	2 (1 - 3)	11 (0.1 - 16.1)	<.0001
	No Long COVID	668	44 (6.6%)	1 (1 - 2)	5.3 (0.1 - 14.3)	202 (30.2%)	2 (1 - 3)	6.3 (0.2 - 15.9)	<.0001

^a^ The number of encounters of each type that happened within 180 days before the index test date was counted for each participant with that type of encounter.

^b^ The total service hours at pre-index period were calculated for each participant with that type of encounter, which were the sum of length in hours of all encounters of each type within 180 days before the index test date.

^c^ The number of encounters of each type that happened between 30–180 days post the index test date was counted for each participant with that type of encounter.

^d^ The total service hours at post-index period were calculated for each participant with that type of encounter, which were the sum of length in hours of all encounters of each type between 30–180 days post the index test date.

^e^ (Q1-Q3) is the range between the first (Q1) and third quartile (Q3).

LC, Long COVID; PAIC, Post-acute Illness Complications.

To calculate the length of each encounter, we applied the Winsorization approach to handle encounters with an atypical length (85th percentile for office and telehealth visits; 95th percentile for hospitalization and emergency visit).

**Fig 3:**
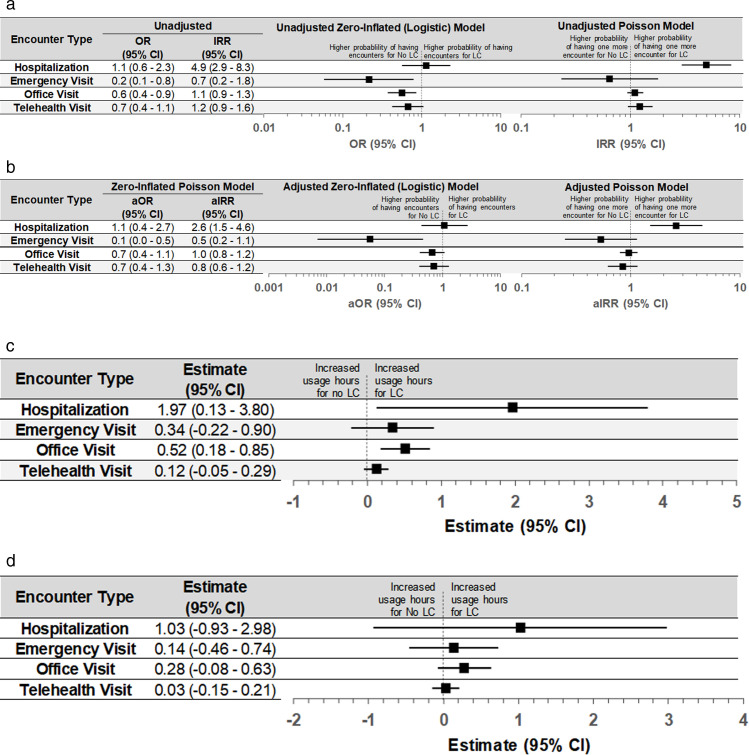
Estimated difference in change of healthcare utilization from pre- to post-index period across the four cohorts. **A, Unadjusted zero-inflated and Poisson model estimates of changes in counts of encounters**. *LC, Long COVID; OR, odds ratio; IRR, incidence-rate ratio.* **B, Adjusted zero-inflated and Poisson model estimates of changes in counts of encounters.** *LC, Long COVID; aOR, adjusted odds ratio; aIRR adjusted incidence-rate ratio.* **C, Unadjusted estimate of changes in length of each encounter**. *LC, Long COVID; CI, confidence interval. Note: To calculate the length of each encounter, we applied the Winsorization approach to handle encounters with an atypical length (85th percentile for office and telehealth visits; 95th percentile for hospitalization and emergency visit).* **D, Adjusted estimate of changes in length of each encounter.** *LC, Long COVID; CI, confidence interval. Note: To calculate the length of each encounter, we applied the Winsorization approach to handle encounters with an atypical length (85th percentile for office and telehealth visits; 95th percentile for hospitalization and emergency visit).*

To examine the number of encounters, we first controlled for the pre-index number of encounters and found that having more pre-index encounters was associated with fewer post-index encounters across all types except emergency department visits. When comparing differences in changes to the number of encounters from pre- to post-index periods across groups, we found a decrease in the odds of having an emergency visits in the LC group compared with the non-LC group (aOR: 0.1; 95% confidence interval [CI] 0.01–0.5), while there were no other significant differences (Fig 3).

Notably, among those with a hospitalization in the pre-index period, there was a nearly three-fold increased risk of hospitalization (adjusted incidence rate-ratio: 2.6; 95% CI 1.5–4.6) among those in the LC group compared with those in the non-LC group (Fig 3).

Overall, the healthcare usage hours increased significantly in the post-index period for both groups when compared with the pre-index period (Table 2, Fig 2). The average length of each encounter was longer for both office visits and hospitalizations in the LC cohort compared with the non-LC cohort (Fig 3). However, after adjusting for baseline differences in populations, the difference was no longer statistically significant (Fig 3).

## Discussion

In this prospective study, we examined healthcare utilization among participants with SARS-CoV-2 infection who developed LC versus those who did not develop LC. Our findings revealed a significant increase in healthcare visits after the acute infection period among both the LC and non-LC cohorts compared with their pre-acute illness period. This may not be a completely unexpected finding, as the pre-index period began in June 2020 when there was more limited access to care due to the combination of public health and other policies to reduce disease transmission particularly in healthcare settings [22–24]. As overall access to care improved over time, this may explain the increase in the pre-index versus post-index visits.

Overall, our unadjusted analyses identified differences in the impact of persistent symptoms on the healthcare system for those with LC. This is consistent with prior work suggesting a higher rate of hospitalization and resource utilization among those with LC [12,13]. However, the majority of these differences diminished after adjustment for age, race, sex and underlying medical conditions, suggesting that baseline characteristics may have influenced this. This difference from prior research may reflect differences in study populations and regional resources, or reliance upon isolated EHR diagnoses in prior studies, whereas our study linked the EHR data with patient-reported information [11–13]. This emphasizes the important consideration of utilizing patient-reported data, as well as accounting for confounders and baseline health to allow a more robust analysis.

Notably, among those who had hospitalizations, the LC group was nearly three times more likely to have additional hospitalizations compared with the non-LC group, which seems less likely to be explained by access or capacity issues. This may reflect a subgroup of those with LC with more severe illness leading to significantly higher rates of re-hospitalization [7]. Alternatively, this may represent other incident illnesses occurring at higher rates or worse severity among those with LC [25,26]. From a public health lens, it is important to consider the potential for repeat hospitalizations in this cohort and allocate more outpatient resources and support systems to reduce the need for inpatient admissions.

Our study also builds upon prior literature by using individual-level EHR data to analyze encounter lengths as a measure of visit complexity between groups. When analyzing differences in visit complexity, we identified longer hospitalizations and office visits in the LC group compared with the non-LC group. Similar to the total visits, this also diminished after adjustment for baseline conditions. This may again be explained by the effect of baseline differences. However, another explanation for these differences may be reduced access to resources and support among those with LC [27]. This may also reflect limited effectiveness of many current interventions for LC.

Notably, nearly half of the entire cohort did not have any office visits in the 180 days after index testing. This may reflect a subpopulation with less severe symptoms, as has been noted in some research [7]. This may also demonstrate continued access issues, which is a particularly critical issue for those suffering from LC.

## Limitations

Our study is limited by requiring that participants had EHR data for at least 180 days before and after their index SARS-CoV-2 test. As such, this excluded participants who did not elect to link their EHR (56% of the initial study cohort) or had incomplete EHR data (17% of the initial study cohort), which could bias the sample. While the overall low rate of utilization may reflect a population with less severe disease, this may also be a strength of our study as this may reflect reduced risk of spectrum bias, thereby enhancing the generalizability of our findings. Moreover, we could only process the EHR data in the Consolidated Clinical Document Architecture (C-CDA) format, and therefore EHR data delivered in other formats was not used by our study, which could bias results if access to and patterns of healthcare vary along with type of EHR across healthcare institutions. As the study relied upon EHR data, we may also have missed visits to clinics that did not have EHR access or were not linked to the participant encounters. Additionally, the study was limited to those who had access to an internet-capable device and completed surveys. Our population also had a higher proportion of people who were White, non-Hispanic, and had a higher annual income compared with the general population. Additional research is needed among those with more social determinants of health vulnerabilities. Moreover, our study was limited to only 180 days post-index; future work should assess outcomes beyond this time period. We were also unable to reliably capture new medical conditions that emerged during the post-index period, so it remains unclear to what degree the higher rate of LC repeat hospitalizations was due to LC symptomatology versus new diagnoses of medical conditions resulting from LC. We utilized a criteria of ≥3 symptoms at 3 months. While there is not a universally-accepted single definition for LC at this time, this definition is consistent with prior work and recent research has shown that this demonstrates a reasonably similar prevalence compared with other common definitions of LC [6,8,16,17,28]. Finally, our study did not assess barriers to care or other potential factors that may have influenced healthcare utilization rates.

## Conclusion

In this prospective study of resource utilization among symptomatic adults with SARS-CoV-2, we found higher rates of repeat hospitalization among those with LC compared with those without LC over the subsequent six months. The total cumulative office visit and hospitalization lengths were longer in the LC cohort, but this difference was no longer significant after accounting for baseline characteristics. These findings demonstrate the healthcare burden resulting from COVID per se and from LC and can inform clinicians and health policy leaders to prepare resources to adequately support patients with LC.

## Supporting information

S1 Table Participant characteristics by electronic health record data availability. (DOCX)

S1 File List of INSPIRE Group members. (DOCX)
