# Supplementary material for: Effects of long COVID on healthcare utilization
Source: PLoS One. 2025 Jul 23;20(7):e0327218. doi: 10.1371/journal.pone.0327218 (PMC12286388; doi:10.1371/journal.pone.0327218)
Supplement: S1 Table — (DOCX) [file pone.0327218.s001.docx]

**S1 Table. Participant characteristics by electronic health record data availability**

| **Participant**  **Characteristics** | **Participants with EHR Data** | | **Participants without EHR Data**  **N=2,269** | ***p-*value** |
| --- | --- | --- | --- | --- |
|  | **Meeting EHR Criteria^a^  (N = 1,115)** | **Not Meeting EHR Criteria^a^  (N = 698)** |  |  |
|  |  |  |  |  |
| **Age^a^** |  |  |  |  |
| Median (Q1-Q3) | 40 (31 – 54) | 36 (28 – 47) | 37 (28 – 50) | <.001 |
| Missing | 0 | 0 | 0 |  |
| **Gender** |  |  |  |  |
| Female | 713 (65.9) | 449 (65.7) | 1492 (67.9) | .27 |
| Male | 345 (31.9) | 220 (32.2) | 676 (30.8) |  |
| Transgender/Non-binary | 24 (2.2) | 14 (2.0) | 29 (1.3) |  |
| Missing | 33 (3.0) | 15 (2.1) | 72 (3.2) |  |
| **Ethnicity** |  |  |  |  |
| Non-Hispanic | 969 (88.2) | 597 (86.8) | 1876 (84.6) | .02 |
| Hispanic | 130 (11.8) | 91 (13.2) | 341 (15.4) |  |
| Missing | 16 (1.4) | 10 (1.4) | 52 (2.3) |  |
| **Race** |  |  |  |  |
| White | 802 (73.6) | 455 (66.9) | 1478 (67.3) | <.001 |
| Black | 105 (9.6) | 62 (9.1) | 162 (7.4) |  |
| Asian | 99 (9.1) | 97 (14.3) | 349 (15.9) |  |
| Other/Multiple | 84 (7.7) | 66 (9.7) | 208 (9.5) |  |
| Missing | 25 (2.2) | 18 (2.6) | 72 (3.2) |  |
| **Education** |  |  |  |  |
| Less than High school | 8 (0.7) | 6 (0.9) | 27 (1.2) | .68 |
| High school graduate | 71 (6.5) | 59 (8.6) | 140 (6.4) |  |
| Some College | 165 (15.0) | 91 (13.2) | 301 (13.7) |  |
| 2-year degree | 75 (6.8) | 47 (6.8) | 153 (7.0) |  |
| 4-year degree | 357 (32.5) | 220 (32.0) | 729 (33.1) |  |
| More than 4 years | 424 (38.5) | 265 (38.5) | 851 (38.7) |  |
| Missing | 15 (1.3) | 10 (1.4) | 68 (3.0) |  |
| **Marital Status** |  |  |  |  |
| Married/partner | 643 (57.9) | 369 (52.9) | 1177 (51.9) | <.001 |
| Divorced/Widowed/Separated | 122 (11.0) | 74 (10.6) | 207 (9.1) |  |
| Never married | 346 (31.1) | 255 (36.5) | 884 (39.0) |  |
| Missing | 4 (0.4) | 0 (0.0) | 1 (0.0) |  |
| **Family Income** |  |  |  |  |
| <10,000 | 63 (5.7) | 42 (6.0) | 124 (5.5) | <.001 |
| 10,000-34,999 | 112 (10.0) | 92 (13.2) | 244 (10.8) |  |
| 35,000-49,999 | 102 (9.1) | 61 (8.7) | 252 (11.1) |  |
| 50,000-74,999 | 149 (13.4) | 87 (12.5) | 305 (13.4) |  |
| ≥75,000 | 641 (57.5) | 377 (54.0) | 1162 (51.2) |  |
| Prefer not to answer | 48 (4.3) | 39 (5.6) | 182 (8.0) |  |
| Missing | 0 (0.0) | 0 (0.0) | 0 (0.0) |  |
| **Health Insurance** |  |  |  |  |
| Private only | 235 (21.1) | 133 (19.1) | 406 (17.9) | .003 |
| Public only | 48 (4.3) | 23 (3.3) | 69 (3.0) |  |
| Private and public | 805 (72.2) | 528 (75.6) | 1700 (74.9) |  |
| None | 27 (2.4) | 14 (2.0) | 94 (4.1) |  |
| Missing | 0 (0.0) | 0 (0.0) | 0 (0.0) |  |
| **Employment** |  |  |  |  |
| Employed | 903 (81.1) | 571 (81.9) | 1848 (81.5) | .89 |
| Not employed | 211 (18.9) | 126 (18.1) | 419 (18.5) |  |
| Missing | 1 (0.1) | 1 (0.1) | 2 (0.1) |  |
| **Tobacco Use** |  |  |  |  |
| Any tobacco use | 129 (11.6) | 94 (13.5) | 337 (14.9) | .03 |
| No tobacco use | 986 (88.4) | 604 (86.5) | 1932 (85.1) |  |
| Missing | 0 (0.0) | 0 (0.0) | 0 (0.0) |  |
| **Comorbidities** |  |  |  |  |
| Asthma | 164 (15.3) | 94 (13.7) | 243 (10.8) | <.001 |
| Hypertension | 196 (18.3) | 92 (13.4) | 262 (11.6) | <.001 |
| Diabetes | 69 (6.4) | 33 (4.8) | 105 (4.7) | .08 |
| Obesity | 324 (30.3) | 182 (26.5) | 574 (25.4) | .01 |
| Emphysema/COPD | 15 (1.4) | 7 (1.0) | 17 (0.8) | 0.20 |
| Heart conditions | 43 (4.0) | 28 (4.1) | 35 (1.6) | <.001 |
| Kidney disease | 22 (2.1) | 9 (1.3) | 21 (0.9) | .03 |
| Liver disease | 15 (1.4) | 3 (0.4) | 17 (0.8) | .07 |
| None | 194 (18.1) | 122 (17.7) | 424 (18.8) | 0.79 |
| I do not know | 176 (16.4) | 166 (24.1) | 551 (24.4) | <.001 |
| Prefer not to answer | 34 (3.2) | 31 (4.5) | 149 (6.6) | <.001 |
| Missing | 44 (3.9) | 10 (1.4) | 11 (0.5) |  |
| **Testing Location** |  |  |  |  |
| At home testing kit | 122 (11.0) | 84 (12.0) | 391 (17.3) | <.001 |
| Clinic including urgent care | 160 (14.4) | 80 (11.5) | 314 (13.9) |  |
| Emergency department | 74 (6.7) | 35 (5.0) | 53 (2.3) |  |
| Hospital | 144 (13.0) | 80 (11.5) | 120 (5.3) |  |
| Other | 71 (6.4) | 49 (7.0) | 200 (8.8) |  |
| Tent/drive up testing site | 537 (48.5) | 370 (53.0) | 1188 (52.4) |  |
| Missing | 7 (0.6) | 0 (0.0) | 3 (0.1) |  |
| **Baseline COVID-19 Vaccination Status^c^** |  |  |  |  |
| Vaccinated | 836 (75.0) | 544 (77.9) | 1,894 (83.5) | <.001 |
| Unvaccinated | 248 (22.2) | 116 (16.6) | 171 (7.5) |  |
| Missing | 31 (2.8) | 38 (5.4) | 204 (9.0) |  |

*EHR, electronic health record*

*^a^ Age was the only continuous variable in this table, which was reported with median and interquartile range (Q1-Q3) and compared across cohorts based on Kruskal-Wallis test.*

*^b^ Hospitalization for index illness was a new question added to the 3-month Follow-up Survey beginning 4-14-2021.*

*^c^ SARS-CoV-2 vaccination status indicates participants with at least one dose prior to the index COVID-19 test; Vaccination initiation information was obtained from linked electronic health record data and patient survey responses.*

*Comorbidity questions were only asked on the 3-month follow-up survey beginning 4-14-2021.*

*All p-values were calculated using chi-square tests except for age.*
